# Outcomes following clonidine ingestions in children: an analysis of poison control center data

**DOI:** 10.1186/s12245-019-0231-1

**Published:** 2019-07-04

**Authors:** Kendra Amico, Rolando Cabrera, Latha Ganti

**Affiliations:** 1 UCF HCA Emergency Medicine Residency Program of Greater Orlando, Orlando, USA; 2 Emergency Medicine and Neurology, University of Central Florida College of Medicine, Orlando, FL USA; 3 Envision Physician Services, Plantation, USA

**Keywords:** Clonidine, Pediatric toxic ingestions, Pediatric overdose

## Abstract

**Background:**

This paper sought to characterize pediatric clonidine ingestions, report trends in incidence, and evaluate outcomes using the Florida Poison Center’s data over a period of 15 years, from 2002 to 2016.

**Results:**

There were 3444 total exposures. Forty percent of the cohort was female. The median age was 5 years. The age distribution changed over time to a higher proportion of teenagers exposed (*p* < 0.0001). From 2002 to 2016, exposures increased from 182 to 378 with a rise in incidence from 4.8 to 9.1 per 100,000 children. Acute on chronic exposures increased from 29.3% to 42.2% (*p* < 0.0001). Female intentional ingestions increased from 52 to 70% (*p* < 0.0001). Twenty-four percent were managed at home, 34% were discharged from the emergency department, 8% were admitted to the floor, and 25% were admitted to the intensive care unit (ICU). Major medical outcomes were associated with older age (*p* = 0.0043, 95% CI 0.0015 to 0.0080) and higher clonidine dose (*p* < 0.0001, 95% CI 0.0347 to 0.0600). Older children were more likely to ingest a larger dose of clonidine (*p* < 0.001, 95% CI 0.0531 to 0.0734), while younger children were more likely to be admitted to the ICU (*p* < 0.001, 95% CI − 0.0092 to − 0.0033). Males were more likely to have acute on chronic ingestions (*p* < 0.001, 95% CI − 0.1639 to − 0.0982); females were significantly more likely to be admitted to the ICU (*p* < 0.0001, 95% CI 0.0380 to 0.0969).

**Conclusions:**

Our analysis shows an increase in the incidence in pediatric clonidine exposures over time despite adjustment for population growth.

## Introduction

Clonidine is a familiar drug readily available in many households. Initially prescribed for adults with hypertension, it has since been applied to other clinical contexts including opioid withdrawal, anxiety, and other psychiatric disorders [1–4]. Clonidine has increasingly been used for behavioral control in pediatric patients particularly since the US Food and Drug Administration (FDA) approval in 2010 for attention deficit hyperactivity disorder (ADHD) in children aged 6–17 years [4–6]. Expanded prescribing and thereby access to this drug has increased the risk for both accidental and intentional ingestions. Emergency providers thus need to be aware of this drug as a potential cause for altered mental status or hemodynamic changes in the pediatric patient. Proper recognition, triage, and disposition of pediatric patients who have accidentally or intentionally overdosed are essential for the emergency medicine provider. By stimulating alpha_2_-adrenergic receptors, clonidine reduces sympathetic outflow from the central nervous system and decreases peripheral resistance, renal vascular resistance, heart rate, and ultimately blood pressure [7, 8]. At times, reactions resemble opioid toxidromes [9]. Commonly experienced adverse reactions include somnolence, fatigue, abdominal pain, and headache [7, 8]. However, clonidine in toxic amounts can cause central nervous system depression and cardiopulmonary instability with features such as apnea, bradycardia, and hypotension [10–12]. Thus it has been included on the “One Pill Can Kill” list for fatal ingestions in pediatric patients [2, 9, 13].

This paper sought to characterize recent trends in incidence of pediatric clonidine ingestions while also evaluating outcomes of pediatric clonidine ingestions.

## Materials and methods

This was a retrospective review of all clonidine ingestions in children aged less than 18 reported to Florida’s Poison Control Centers from January 1, 2002, through December 31, 2016. Over the study interval, the number of poison control centers in Florida remained stable at three. The total pediatric population of Florida grew by 9.86% over the years 2002–2016 with an annual growth rate of 0.70% [14].

Patients were followed for a minimum of 24 h. Exposures were characterized by intentionality (“unintentional” versus “intentional”), polydrug versus isolated clonidine ingestion, and acuity (“acute” if the event was a one-time ingestion versus “acute on chronic” if the patient was previously receiving long-term clonidine therapy and overdosed on his own medication). Total clonidine ingestion when available was listed. Medical outcomes were classified as minor (symptoms that were minimal and resolved rapidly) and major (symptoms that were systemic, required treatment, or were life-threatening). Disposition was characterized as managed “on site” (home or a non-healthcare facility), discharged from the emergency department (ED), admitted to a medical floor, admitted to a psychiatric facility, or admitted to an intensive care unit (ICU).

The primary outcome of this study sought to characterize the frequency and incidence rate of pediatric clonidine ingestions in Florida over a period of 15 years. Children with an exposure to Catapres® (Manufacturer: Boehringer Ingelheim Pharmaceuticals, Ridgefield, CT, USA), Catapres-TTS® (Manufacturer: Boehringer Ingelheim Pharmaceuticals, Ridgefield, CT, USA), and Kapvay® (Manufacturer: Concordia Pharmaceuticals Inc., Oakville, Ontario, Canada) were included. Secondary outcomes included description of the exposures in relation to age, sex, dose, intentionality, acuity of exposure, medical outcomes, and disposition.

Poison control data were collected through the Toxic Exposure Surveillance System (TESS). The authors would like to acknowledge the Florida Poison Information Center-Tampa for their assistance in providing the TESS data for analysis. Data were abstracted onto a predesigned standardized data collection instrument by personnel blinded to the outcomes of interest. Descriptive statistics and data analysis using multivariate regression analysis were performed using Excel.

The Institutional Review Board at the University of Central Florida determined that this study met criteria for exempt status.

## Results

Three thousand four hundred forty-four potentially toxic clonidine ingestions were reported to the Florida’s Poison Control Centers throughout the 15 years examined (Table 1). Forty percent of the cohort was female. Further, 50.6% were less than 6 years old, 28.0% ages 6–11, and 21.3% were greater than 11 years of age. The median age was 5 years (interquartile range (IQR) 2 to 10). The age distribution changed over time to an increasing proportion of teenagers exposed from 19.7% in 2002 to 32.2% in 2016 (*p* < 0.0001, *z* test for proportions).

**Table 1:** Florida pediatric clonidine ingestions from 2002 to 2016 by age group

	Age	< 6 (*n* = 1744)	6–11 (*n* = 966)	12–17 (*n* = 734)
	Total exposures (*n* = 3444)	1744 (50.6)	966 (28.0)	734 (21.3)
Gender	Male (*n* = 2077)	982 (47.3)	725 (34.9)	370 (17.8)
	Female (*n* = 1363)	760 (55.8)	239 (17.5)	364 (26.7)
Disposition	Discharged from ED (*n* = 1180)	722 (61.2)	290 (24.6)	168 (14.2)
	Medical admission (*n* = 276)	158 (57.2)	56 (20.3)	62 (22.5)
	ICU admission (*n* = 854)	548 (64.2)	99 (11.6)	207 (24.2)
	At home management (*n* = 834)	213 (25.5)	465 (55.8)	156 (18.7)
	AMA (*n* = 184)	102 (55.4)	49 (26.6)	33 (17.9)
	Psychiatry admission (*n* = 115)	1 (0.9)	7 (6.0)	107 (93.0)
	Death (*n* = 0)	0	0	0
Outcome	Minor (*n* = 2196)	1115 (50.8)	664 (30.2)	417 (19.0)
	Major (*n* = 888)	492 (55.4)	147 (16.6)	249 (28.0)
	Unknown (*n* = 360)	137	155	68
Acuity	Acute (*n* = 2161)	1503 (69.6)	338 (15.6)	320 (14.8)
	Acute on chronic (*n* = 1258)	238 (18.9)	624 (49.6)	396 (31.5)
	Unknown (*n* = 25)	3 (12.0)	4 (16.0)	18 (72.0)
Ingestion type	Clonidine only (*n* = 2540)	1388 (54.6)	726 (28.6)	426 (16.8)
	Mixed (*n* = 904)	356 (39.4)	240 (26.5)	308 (34.1)
Intentionality	Accidental (*n* = 2889)	1732 (60.0)	887 (30.7)	270 (9.3)
	Intentional (*n* = 534)	6 (1.1)	72 (13.5)	456 (85.4)
	Unknown (*n* = 21)	6 (28.6)	7 (33.3)	8 (38.1)

Data are reported as *n* (%)

*ED* emergency department, *ICU* intensive care unit, *AMA* against medical advice

**Fig. 1:**
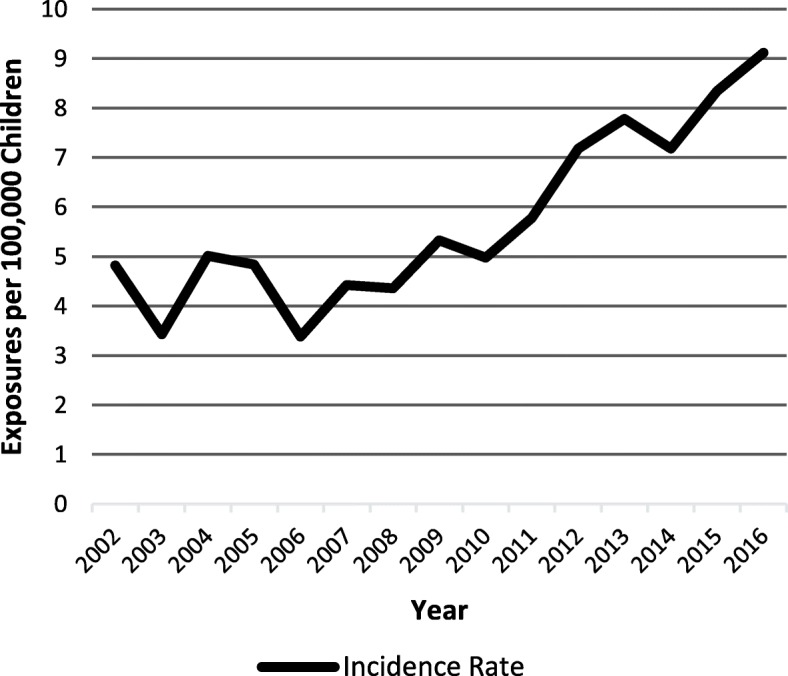
Incidence rate over time

**Fig. 2:**
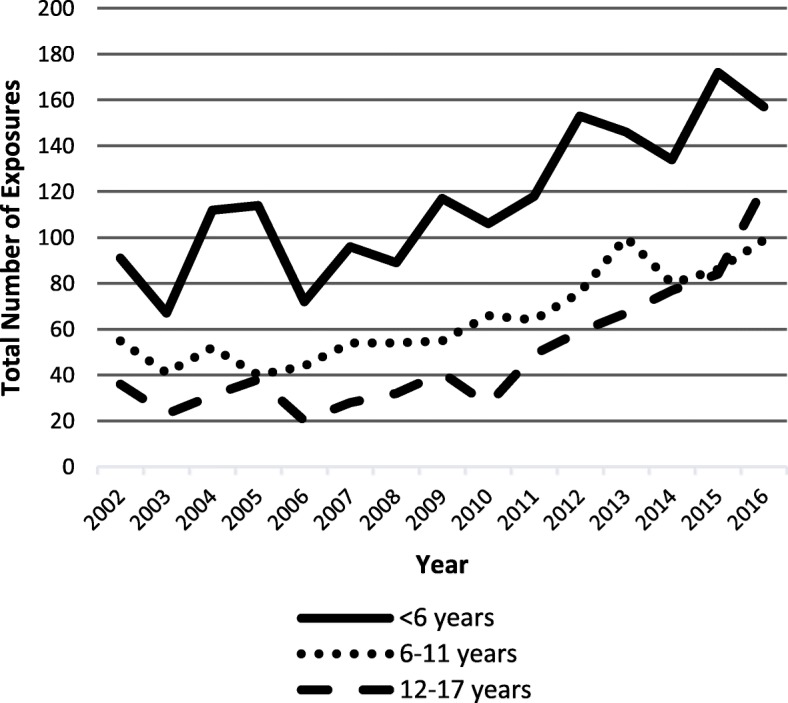
Clonidine exposure over time by age

Total clonidine exposures increased from 182 to 378 exposures (range 131–378) over the study period. Similarly, the incidence rate increased from 4.8 per 100,000 children in 2012 to 9.12 per 100,000 children in 2016 (Fig. 1). When examined in age groups of < 6, 6–11, and 12–17 years, an increase in clonidine exposures over time was still observed separately in all groups (Fig. 2).

Moreover, 36.5% of total patients overdosed on their own medications; when examined by age, clonidine was the child’s own medication in 13.6%, 64.0%, and 54.0% of children < 6, 6–11, and > 11 years of age respectively. Over the study period, there was an increase in the exposures deemed to be acute on chronic from 29.3% in 2002 to 42.2% in 2016 (*p* < 0.0001, *z* test for proportions)

Intentional overdoses accounted for 15.5% of total exposures versus 0.3%, 7.0%, and 62.0% of exposures by < 6, 6–11, and > 11 year olds respectively. While total exposures were consistently higher in males, the percentage of intentional ingestions by female patients increased over time from 52% in 2002 to 70% in 2016 (*p* < 0.0001, *z* test for proportions).

**Fig. 3:**
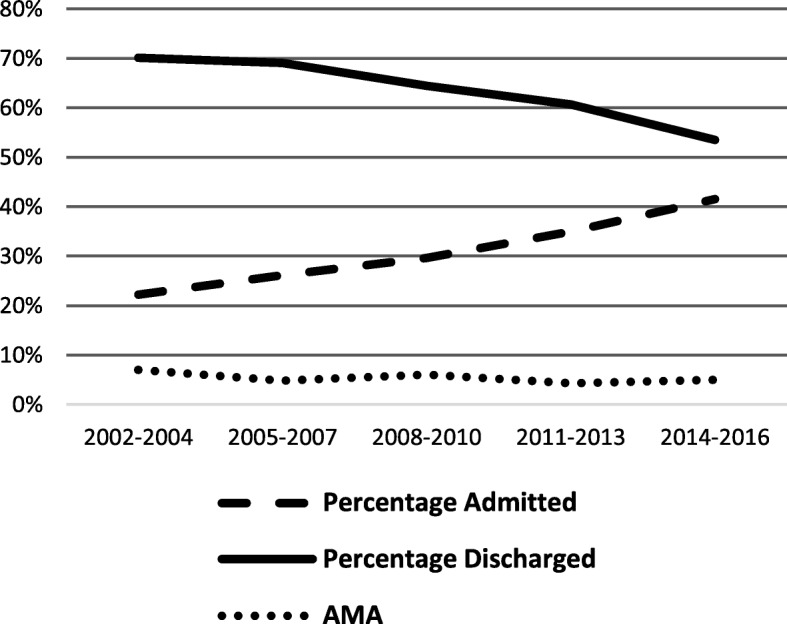
Disposition trend over time

In terms of disposition, 24% were managed at home, 34% were seen in the emergency department and discharged, 8% were admitted to the floor, 25% were admitted to the intensive care unit (ICU), 3% were admitted to psychiatry, and 5% left against medical advice (AMA) or were lost to follow-up. Over time, the percentage of patients admitted to an inpatient setting increased (Fig. 3).

There were no deaths reported during this timeframe. Within the cohort, 36.2% had a major medical outcome. Major medical outcomes were associated with older age (*p* = 0.0043, 95% CI 0.0015 to 0.0080) and higher clonidine dose (*p* < 0.0001, 95% CI 0.0347 to 0.0600). Polydrug versus clonidine-only ingestion and exposure acuity were not statistically significant. The median clonidine dose was 0.2 mg (IQR of 0.1 to 0.5, range of 0.025 to 27.5) for the entire population. Older children were more likely to ingest a larger dose of clonidine (*p* < 0.001, 95% CI 0.0531 to 0.0734): median dose ingested for children less than 6 years was 0.2 mg compared to 0.5 mg for those over 11 years of age. Then, 31.4%, 10.2%, and 28.2% of children less than 6, 6–11, and greater than 11 years of age respectively were admitted to the ICU. However, when considering the entire population, younger children were statistically more likely to be admitted to the ICU (*p* < 0.001, 95% CI − 0.0092 to − 0.0033).

Males were more likely to have acute on chronic ingestions (*p* < 0.001, 95% CI − 0.1639 to − 0.0982): 40.6% of males versus 28.5% of total female ingestions. Females were significantly more likely to be admitted to the ICU (*p* < 0.0001, 95% CI 0.0380 to 0.0969): 22.1% of total male compared to 28.9% of total female ingestions.

## Discussion

There have been numerous case reports and case series but very few larger studies examining pediatric clonidine ingestions were published in the literature [11–15]. The study described in this paper adds to the limited data available on this topic; and to our knowledge, is the longest study period to date, examining exposures over a 15-year span. This allowed for examination of trends and changes in outcomes over time related to pediatric clonidine overdoses.

Our analysis shows an increase in the incidence in pediatric clonidine exposures over time when adjusted for population growth; this is similar to a previous study which demonstrated a rise in the number of exposures from 1993 to 1999 [15]. In our study however, there was a relatively abrupt increase in the incidence rate around 2010 which may be partially attributable to change in prescribing patterns [3]. This may also explain the increase in acute on chronic exposures over time, as later on in the study period, children were more likely to overdose on their own medications. The increase in clonidine ingestions parallels an increase in clonidine prescribing which has been consistently documented in the literature, even preceding FDA approval of this drug for ADHD [16, 17]. A study by Zito et al. looked at statewide clonidine utilization among preschool Medicaid enrollees aged 2–4 years old and noted a 28-fold increase in the prevalence rate per 1000 from 0.1 to 2.3 during 1991 to 1995 [6]. Another study demonstrated that from 2003 to 2008, the proportion of Medicaid children 3–18 years old receiving clonidine nearly doubled across all age groups [18]. Currently, clonidine usage in very young children, those 5 years and younger, still constitutes off-label prescribing, which raises concerns due to the limited safety and efficacy data in this specific population [5, 19].

Clonidine ingestions can lead to serious adverse effects [8, 10, 13]. Our study had no deaths related to clonidine exposure, similar to other published observational and case reports [20–22]. Clonidine-associated pediatric fatalities have been rarely reported in the literature: the study by Klein-Schwartz reported one fatality out of 10,060 exposures [15]. While mortality is rare, the significant morbidity caused still results in a need for hospitalization, invasive monitoring, and mechanical ventilation [12, 23]. Still, 24% of our patient population was managed at home. This reflects current Florida Poison Center standardized recommendations which indicate a patient may be monitored at home if the patient is asymptomatic and has consumed a quantity of clonidine less than 0.1 mg for children < 5 years of age, 0.2 mg for children 5–8 years, < 0.4 mg for children 9 and older. For an ingestion in asymptomatic children who was regularly taking this medication, referral to a hospital setting was not indicated if the child had ingested less than double their therapeutic or prescribed daily dose.

In our study, the proportion of patients admitted to a medical floor or ICU increased over time. Twenty-five percent were admitted to the ICU which was slightly higher compared to 20% reported in a similar study from the 1990s [15]. Younger patients were more likely to be admitted to the ICU, which likely reflects the higher proportion of young children in the study population. This may also be related to the unreliable effects of clonidine ingestion [24]. In our study, higher clonidine dose was associated with worse outcome. While some studies have attempted to establish a dose-response relationship, there is no clear weight-based dose at which clonidine is toxic or lethal [12, 22, 25]. Thus, this may prompt physicians to practice conservatively and admit younger patients to a higher care setting.

While we included all known reported clonidine ingestions during the study time period, data were limited to cases called in to poison control. If individuals or providers chose not to call, these data would be lost. Some patients were lost to follow-up which may limit outcome results; in our study sample, this was 5.3%. The dataset does not differentiate short-acting vs. extended-release clonidine which could affect outcomes. Also, outcomes may be affected in the setting of polypharmacy ingestion; however, our study found no relationship between polydrug versus clonidine-only ingestions and outcomes. We studied only clonidine ingestions and did not include ingestions of guanfacine, a similar agent. Guanfacine is a newer drug and thought to be less sedating compared to clonidine which may affect prescriber patterns; it received FDA approval for ADHD of pediatric patients in 2009, similar to clonidine [26].

## Conclusion

This study demonstrated an increase in the incidence of pediatric clonidine accidental and intentional ingestions over the study period. Given clonidine’s high yet unpredictable potential for neurologic and cardiac effects, these patients are being increasingly admitted to a monitored setting.

## Abbreviations

ADHD: Attention deficit hyperactivity disorder
ED: Emergency department
EMS: Emergency medical services
FDA: Food and Drug Administration
